# Supplementary material for: Empirical methods for controlling false positives and estimating confidence in ChIP-Seq peaks
Source: BMC Bioinformatics. 2008 Dec 5;9:523. doi: 10.1186/1471-2105-9-523 (PMC2628906; doi:10.1186/1471-2105-9-523)
Supplement: Additional File 1 — A variety of html documents from the USeq web site detailing the available applications, their best usage, output file type descriptions, command line menus, etc. [file 1471-2105-9-523-S1.zip › USeqUserGuides/JavaDoc.zip!/JavaDoc/bioroot/package-summary.html]

bioroot


|  |  |  |
| --- | --- | --- |
| **Overview** \| **Package** \| Class \| **Use** \| **Tree** \| **Deprecated** \| **Index** \| **Help** | |  |
| PREV PACKAGE   **NEXT PACKAGE** | **FRAMES**    **NO FRAMES**     **All Classes** |  |


---

## Package bioroot

| **Class Summary** | |
| --- | --- |
| **AccountManagement** |  |
| **Archive** |  |
| **BlastMatcher** |  |
| **CellType** |  |
| **CellTypeBean** |  |
| **Contact** |  |
| **ContactBean** |  |
| **CronArchive** | Emails lab group's collections to superusers. |
| **DBUtil** | Utility class to hold database relevant methods and fields, some from Hall and Brown The following is a collection of stuff for interacting with a SQL database First open a connection by calling connectMySQL, then get results with executeSQL, then close the connection with closeConnection. |
| **DeleteMeNow** |  |
| **Feature** |  |
| **Gene** |  |
| **GeneBean** |  |
| **IdNameMatch** |  |
| **LabGroup** |  |
| **Login** |  |
| **LoginBean** |  |
| **Logout** | Logout page, kills session, frees objects |
| **Marker** |  |
| **MarkerBean** |  |
| **Organism** |  |
| **OrganismBean** |  |
| **Organization** | Container for holding info about Organization membership. |
| **PatternMatch** | Class to hold information and helper methods relating to pattern matches. |
| **Preference** |  |
| **ReagentBean** |  |
| **Search** |  |
| **SearchBean** | Bean to hold info about a search request |
| **SearchHit** | Holds info relating to a search hit from a database LIKE query. |
| **Spammer** | For sending out email messages |
| **TableHeading** |  |
| **UserBean** | User information. |
| **UserRegistration** |  |
| **UserRegistrationBean** |  |
| **Util** | Utility class for BioRoot |

---


|  |  |  |
| --- | --- | --- |
| **Overview** \| **Package** \| Class \| **Use** \| **Tree** \| **Deprecated** \| **Index** \| **Help** | |  |
| PREV PACKAGE   **NEXT PACKAGE** | **FRAMES**    **NO FRAMES**     **All Classes** |  |


---
